# 

**Published:** 1885-04-20

**Authors:** 


### TABLE OF CONTENTS.

ORIGINAL AND SELECTED ARTICLES:

Successful Treatment of Cancer with Arsenic, by J. McF. Gaston, M.D., Professor of Principles and Practice of Surgery in the SOUTHERN MEDICAL COLLEGE, Atlanta, Georgia, 121. A Case of Rupture of a Muscular Attachment Followed by a Large Abscess, by W. A. Crow, M.D., Friendship, Va., 125. Dugas' Bandage for the Breast, by T. L. Lallerstedt, M.D., of Georgia, 126. Antisepsis in Vienna, 127. Typho-Malarial Fever, 130. Wound Dressing: Iodoform Intoxication, 133. On Stone in the Female Bladder, 135. How and When to Deliver the Placenta, 137.

ABSTRACTS AND GLEANINGS:

Cocaine in Sleeplessness; The External Use of Chloroform in Labor, 140. The Treatment of Intra-Uterine Diseases; Calomel in the Treatment of Otorrhœa, 141. Hypodermic Injections of Morphia in Convulsions, 142. A Case of Gasric Ulcer, 143. Cocaine in Minor Surgery; Treatment of Gonorrhœa: Diabetes, 144. Can the Sex of the Child be Determined Before Birth; An Unintentional Pun, 145. The Cure of Asthma; Electricity in Obstetrics; Ergot in Treatment of Dysentery, 146. Hay Fever and its Successful Treatment; A New Treatment for Neuralgia; Opium Habit, 147. Alvelos: A New Cure for Cancer; The Aspirator in Hydrothorax; The Simaba Cedron as a Remedy for Hydrophobia; Phenylhydrazine, 148. Amblyopia; Permanganate of Potassium in Amenorrhœa; Cannabis Indica as a Local Anæsthetic; Jacaranda Lancifoliata; The Muriate of Erythroxylon, 149. Jaborandi in Obstinate Hiccough; Twins Born Four Days Apart; Reduction of Dislocation of the Humerus; The Treatment of Albuminuria with Chloral; Perosmic Acid in Sciatica, 150.

SCIENTIFIC ITEMS:

The Microphotoscope, 151. A Great Animal; Telescopes; Platinum Crucibles, 152.

PRACTICAL NOTES AND FORMULÆ.

A Good Fever Mixture; Counter-Irritants; Improved Formula for Syrup of Dover's Powder, 153. Chronic Ague; Nurse's Sore Mouth; Pruritus Vulvæ; Hypodermic Injection of Apomorphine; Extemporaneous Solution of Hydrobromate of Quinine; Glycerite of Alum, 154.

EDITORIALS AND MISCELLANEOUS.

Notice to Subscribers in Arrears; Editorial Notices, 155. Cancer; Death of Dr. Few, of South Carolina; Charge to the Graduating Class of the SOUTHERN MEDICAL COLLEGE, March 4th, 18[illegible]5, by Professor Thos. S. Powell, M.D., 156. Books and Pamphlets Received, 159. Receipted; Special Notices, 160.

